# Novel post-translational modifications in fibrosis: The mechanisms and implications

**DOI:** 10.1016/j.gendis.2026.102194

**Published:** 2026-04-17

**Authors:** Qianshi Wu, Li Luo, Yanqun Li, Jidi Wu, Junle Li, Wanhong He, Yulin Mou, Shiying Qu, Yanqi Lei, Wei Huang, Ling Cao

**Affiliations:** a Department of Nephrology, The Affiliated Hospital of Southwest Medical University, Luzhou, Sichuan 646000, China; b Department of Endocrinology and Metabolism, The Affiliated Hospital of Southwest Medical University, Luzhou, Sichuan 646000, China; c Sichuan Clinical Research Center for Diabetes and Metabolic Diseases, Luzhou, Sichuan 646000, China; d Metabolic Vascular Disease Key Laboratory of Sichuan Province, The Affiliated Hospital of Southwest Medical University, Luzhou, Sichuan 646000, China; e Sichuan Clinical Research Center for Nephropathy, Luzhou, Sichuan 646000, China; f The Second People's Hospital of Deyang, Deyang, Sichuan 618000, China

**Keywords:** Antifibrotic agents, Epigenetics, Fibrosis, Histone modification, Post-translational modifications

## Abstract

Fibrosis constitutes a pathological phenomenon characterized by excessive deposition of fibrous connective tissue and concomitant reduction of parenchymal cells in tissues and organs. Post-translational modifications (PTMs: chemical modifications of proteins after their synthesis, regulating protein function), including phosphorylation, ubiquitination, and acetylation, encompass biochemical processes occurring following ribosomal protein synthesis, involving covalent conjugation of functional groups to polypeptide chains. Beyond well-characterized modifications, novel PTMs such as propionylation, butyrylation, crotonylation, malonylation, succinylation, glutarylation, and lactoylation are increasingly recognized. While classical and traditional PTMs have received considerable research attention in fibrogenesis, contemporary investigations are elucidating the pathological contributions of novel PTMs to fibrosis progression. This review highlights how novel PTMs contribute to fibrosis progression and discusses their potential as therapeutic targets for anti-fibrotic interventions.

## Introduction

Fibrosis represents a pathological hallmark of diverse systemic pathologies, encompassing idiopathic pulmonary fibrosis, hepatic cirrhosis, and cystic fibrosis. These fibrotic disorders constitute a major global health burden, incurring considerable healthcare expenditure through disease management and lost productivity. Hepatic cirrhosis is the ninth leading cause of male disability-adjusted life-years within the top decile of level 3 disease burdens according to the epidemiological data from the 2019 Global Burden of Disease Study.^1^ Collectively, fibrotic conditions exert a substantial public health impact, with prevalence estimates suggesting their involvement in 25% of global mortality cases and annual incidence rates of major fibrotic disorders reaching 1 in 20 individuals.^2^

Proteins constitute primary biological effectors whose structural dynamics and functional capacities are precisely modulated through post-translational modifications (PTMs). Histone PTMs primarily occur on the conserved flexible N-terminal tails of core histones and linker histone, and are dominated by lysine/arginine-targeted modifications. Some of these modifications exhibit conserved epigenetic signatures and are bidirectionally regulated by site-specific writers and erasers.^3^, ^4^, ^5^ By contrast, non-histone PTMs encompass a much broader spectrum of modification types, which target diverse amino acid residues distributed throughout the entire sequence of functionally heterogeneous proteins. Beyond epigenetic regulation, their biological functions directly modulate protein intrinsic activity, subcellular localization, protein–protein interactions, and signal transduction cascades, with the functional outcomes of these modifications tightly dependent on the specific properties of target proteins and the cellular microenvironment.^6^ Among the classic PTMs, phosphorylation, methylation, and SUMOylation have been extensively investigated for their pivotal regulatory roles. Phosphorylation, catalyzed by kinases and reversed by phosphatases, predominantly targets serine, threonine, or tyrosine residues, acting as dynamic molecular switches that govern signal transduction cascades and protein–protein interactions.^7^ Methylation, mediated by methyltransferases and demethylases, modifies lysine or arginine residues in both histones and non-histones; its mono-/di-/trimethylation states can encode specific epigenetic signals or modulate transcription factor activity.^8^ SUMOylation, a ubiquitin-like modification involving an E1-E2-E3 enzyme cascade, covalently conjugates small ubiquitin-like modifier proteins to lysine residues of target proteins, thereby regulating the subcellular localization, stability, and functional involvement of these targets in DNA damage repair and transcriptional regulation.^9^ Recent advances in mass spectrometry-driven proteomics have catalyzed the discovery of non-canonical PTMs,^10^ notably lactylation,^11^ palmitoylation,^12^ crotonylation,^13^ myristoylation,^14^ β-hydroxybutyrylation,^15^ malonylation,^16^ and succinylation.^17^ These novel modifications have become focal points of research, as investigators have linked their aberrant states to various pathophysiological conditions, including fibrosis.

Single-cell genomic advancements now enable multi-omics interrogation of fibrotic mechanisms through integrated genomic–proteomic analyses. Experimental evidence demonstrates that dynamic modulation of histone/DNA methylation and acetylation states differentially regulates fibrogenic signaling cascades, either propagating or attenuating fibrotic progression.^18^ Traditional investigations have predominantly focused on canonical PTMs, including acetylation, methylation, and ubiquitination. This review synthesizes emerging evidence linking non-canonical PTMs to fibrogenesis, while mechanistically contextualizing their pathophysiological roles within technological advancements. The therapeutic potential of these PTM-directed mechanisms is critically evaluated, proposing novel intervention strategies for fibrotic disorders.

## Overview of fibrosis mechanisms

Fibrosis constitutes a maladaptive reparative response to organ/tissue injury, characterized by persistent fibroblast activation and excessive deposition of extracellular matrix (ECM) components, particularly collagens I/III and fibronectin isoforms. This pathological remodeling manifests as progressive parenchymal atrophy concurrent with fibrous connective tissue accumulation, demonstrating aberrant architectural remodeling. As a progressive and typically irreversible process, fibrogenesis drives disproportionate matrix accumulation over parenchymal regeneration, culminating in structural/functional compromise across multiple organ systems. Current mechanistic research prioritizes deciphering the pathobiological drivers of fibrogenesis to develop rational therapeutic strategies against this major contributor to global disease burden.

Fibrotic pathogenesis is fundamentally characterized by myofibroblast activation, which drives excessive ECM (a macromolecular network secreted by cells, with abnormal accumulation often observed in pathologies like fibrosis) deposition and functions as a central mediator of fibrotic progression.^19^^,^^20^ The transforming growth factor-beta (TGF-β: a multifunctional cytokine regulating cell growth, differentiation, and tissue repair/fibrosis) signaling cascade constitutes a central pathway in fibrogenesis. TGF-β triggers canonical signaling through transmembrane receptor kinase activation. Receptor activation induces phosphorylation of downstream Sma and Mad-related protein (Smad: key mediators in TGF-β signaling, transmitting signals to the nucleus) effector proteins. Notably, Smad2/3 serve as principal signal transducer molecules mediating signal transmission. Following Smad4 binding, the heterotrimeric complex translocates to the nucleus to orchestrate fibrogenic gene expression.^21^^,^^22^ TGF-β drives fibrogenesis by promoting myofibroblast transdifferentiation, stimulation of excessive ECM biosynthesis, and impairment of matrix turnover.^23^, ^24^, ^25^, ^26^ Furthermore, TGF-β synergizes with Wnt/β-catenin signaling through pathway crosstalk, exacerbating fibrotic outcomes.^27^^,^^28^ TGF-β orchestrates pleiotropic effects via interaction with complementing pathways, including Notch,^29^ mitogen-activated protein kinase (MAPK: a kinase that transmits extracellular signals to regulate cell proliferation, differentiation, etc.),^30^ and nuclear factor kappa B (NF-κB: a key transcription factor regulating genes for inflammation, immunity, and cell survival),^31^ propagating fibrotic pathogenesis. These molecular interactions provide novel mechanistic insights and druggable targets for anti-fibrotic therapeutic development.

The fibrosis pathobiology and therapeutic innovation remain prioritized research domains in translational medicine. This review systematically examines PTM-mediated regulatory networks as novel mechanistic determinants and therapeutic vulnerabilities in fibrogenesis.

## Post-translational modifications in fibrosis

### Lactylation

The seminal 2019 *Nature* study by Zhang et al identified lactate-driven protein lactylation as a novel post-translational modification, initiating the formal investigation of lactylation as a regulatory mechanism.^3^ This discovery fundamentally transformed the understanding of lactate-mediated signaling, particularly through histone-specific modification. Emerging evidence shows that spatiotemporal lactate dynamics are precisely regulated via concentration gradients, lactate dehydrogenase activity, and delactylase function.^32^ In addition, lactylation modifications are not limited to histones but also extend to affecting the function of kinases and transcription factors.^33^

With the deepening of research into lactylation, it has been found that lactylation exists and plays an important role in various disease processes.^34^, ^35^, ^36^, ^37^, ^38^, ^39^, ^40^, ^41^ The relationship between lactylation and fibrosis is indeed a prominent area of research. The study found that Snail1 undergoes lactylation modification, thereby activating the TGF-β/Smad2 signaling pathway to promote endothelial-to-mesenchymal transition (endothelial cells converting to mesenchymal cells, gaining migratory/invasive abilities), and ultimately exacerbating cardiac fibrosis and dysfunction after myocardial infarction. This leads to a decrease in the left ventricular ejection fraction of mice by 14.0% and 19.5% at 7 and 28 days, respectively, and a reduction in fractional shortening by 15.8% and 21.6% at 7 and 28 days, respectively.^42^ Similarly, placental hypoxia drives lactate accumulation, which enhances H3K18la to up-regulate the expression of fibrosis-related genes fibronectin (*FN*: an ECM protein mediating cell adhesion and matrix assembly) and serpin family E member 1 (*SERPINE1*: a serpin inhibiting plasminogen activators, regulating fibrinolysis/matrix), ultimately leading to ECM deposition in the placental villous stroma and placental fibrosis.^43^

In the kidneys of mouse models of ischemia–reperfusion injury (tissue damage caused by reperfusion after ischemia, exacerbating injury), H4K12 lactylation promotes renal fibrosis by activating the NF-κB pathway;^44^ similarly, H3K18la can drive renal fibrosis in diabetic kidney disease,^45^ while in mouse models of acute kidney injury, lactylation of H3K18 and Ezrin may contribute to renal fibrosis via activation of the RhoA/ROCK pathway.^46^^,^^47^

Furthermore, two articles have demonstrated from different perspectives that histone lactylation induces pro-fibrotic activation of macrophages, thereby contributing to pulmonary fibrosis.^48^^,^^49^ In addition, in the context of liver fibrosis, researchers have also identified lactylation induction. Rho et al have demonstrated that hexokinase 2 (HK2)-mediated H3K18 lactylation is essential for liver fibrosis progression.^50^ Huang et al have shown that excessive lactate accumulation in skeletal muscle of overexercised mice induces SORBS3 lactylation, which promotes hepatocyte lactate uptake and liver fibrosis via the MCL-BAX/BAK pathway.^51^ Separately, lactylation modulates the deubiquitinating activity of the deubiquitinase ubiquitin-specific protease 2 (USP2) via PTM, thereby forming a “lactylation–USP2–substrate ubiquitination” crosstalk axis that ultimately mediates organ fibrosis.^52^

Lactylation, an epigenetic modification driven by lactate accumulation, acts as a key driver of fibrosis^53^ (Fig. 1). Targeting lactylation holds promise for antifibrotic therapy. Small molecules like lactate dehydrogenase (LDH) inhibitors and delactylase activators can modulate lactylation. For instance, sirtuin 5 (SIRT2) functions as a delactylase that inhibits the activity of USP2 to ameliorate fibrosis.^52^ Other agents, such as liraglutide, have been found to block p300 lactylation, inhibit fibroblast activation, and ultimately alleviate pulmonary fibrosis.^54^ However, all relevant studies remain in the preclinical stage. Notably, since lactylation is involved in the metabolic regulation of normal cells, targeted interventions may disrupt physiological homeostasis. This can trigger potential side effects, including glycemic disorders and aberrant cellular energy metabolism, thus necessitating the precise targeting of fibrotic tissues or specific modification sites to mitigate such risks.

**Figure 1:**
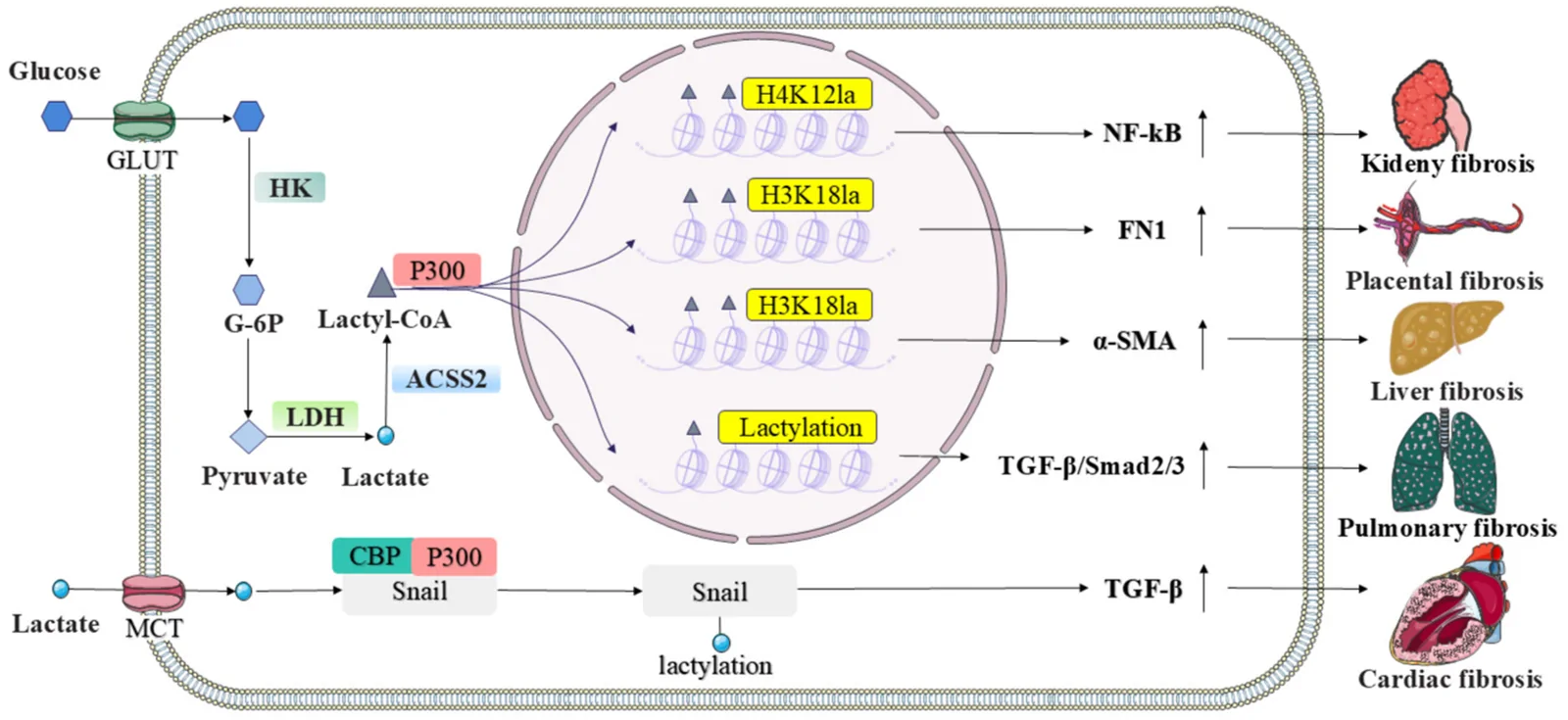
Role and mechanism of lactoylation in fibrotic diseases. Glucose enters the cell and undergoes glycolysis in the cytoplasm to form lactate. After being acted upon by ACSS2, it becomes lactyl-CoA. After entering the nucleus, it promotes lactylation of different histone sites, thus affecting the expression of target genes and eventually leading to tissue and organ fibrosis. Additionally, lactate entering the cytoplasm can cause lactylation of non-histone proteins, which is associated with cardiac fibrosis. GLUT, glucose transporter; MCT, monocarboxylate transporter; HK, hexokinase; G-6P, glucose 6-phosphate; LDH, lactate dehydrogenase; α-SMA, α-smooth muscle actin.

However, current PTM detection techniques have notable limitations, including insufficient sensitivity for low-abundance PTMs, the inability to precisely distinguish the functional differences between distinct PTM modification sites, and complex, high-cost detection workflows.^55^ These drawbacks hamper in-depth investigations into novel PTMs such as lactylation. Additionally, critical knowledge gaps persist regarding PTM crosstalk networks, the identification of organ-specific lactylation targets, and the upstream and downstream molecular interactions of regulatory pathways.^56^ Future research addressing these gaps will accelerate the development of lactylation-targeted therapeutics and their clinical translation for fibrotic disorders.

### Palmitoylation

From the perspective of palmitoyltransferases and depalmitoylases, a study demonstrated that both protein palmitoylation levels and the expression of the key palmitoyltransferase DHHC9 are significantly down-regulated in fibrotic kidney tissues of mouse models and clinical samples from patients with chronic kidney disease.^57^ Specifically, DHHC9 catalyzes the palmitoylation of β-catenin at the cysteine 300 residue, and this modification further promotes the ubiquitination and degradation of β-catenin. Ultimately, DHHC9 exerts a renoprotective effect against renal fibrosis by inhibiting the synthesis and deposition of fibronectin (Fig. 2). Moreover, artemether alleviates liver fibrosis by inducing hepatic stellate cell ferroptosis via DHHC12-mediated Beclin 1 (BECN1) protein S-palmitoylation.^58^ Within the spectrum of multi-organ fibrotic diseases, cystic fibrosis is characterized by abnormal mucus secretion affecting multiple organs, and its pathological process is closely linked to fibrosis of the lungs, liver, pancreas, and intestines. The core pathogenesis of cystic fibrosis lies in the maturation defect of the cystic fibrosis transmembrane conductance regulator (CFTR), a transmembrane ion channel that regulates chloride transport. Palmitoylation plays a pivotal role in the maturation and membrane trafficking of CFTR, and its functional abnormalities can indirectly drive disease initiation and progression^59^ (Fig. 3), suggesting an intrinsic link between the pathological mechanisms of cystic fibrosis and the regulatory network of palmitoylation. In addition, dysregulated palmitoylation has been confirmed to be closely associated with the pathogenesis of hepatic fibrosis^60^ and systemic sclerosis.^61^ Taken together, palmitoylation is not a consequence of fibrosis but a key factor during the fibrotic process.

**Figure 2:**
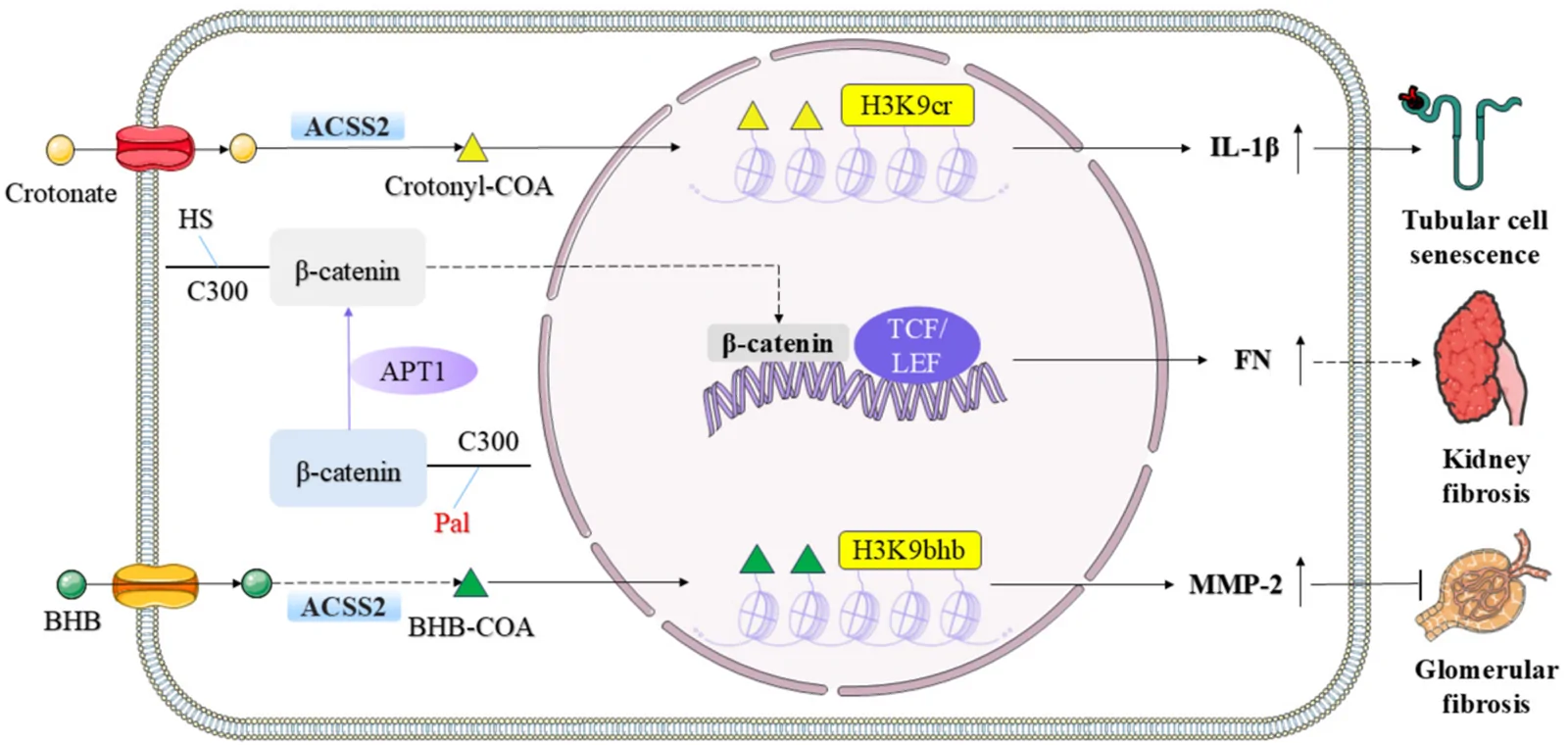
Crotonylation, β-hydroxybutyrylation, and palmitoylation in kidney fibrosis. Crotonate and β-hydroxybutyrate (BHB) enter the cytoplasm and may be converted into corresponding Coenzyme A (CoA) by ACSS2, then enter the nucleus to modify certain histone sites, eventually leading to renal tubular and glomerular fibrosis. Meanwhile, palmitoylation of β-catenin at Cys300 inhibits activation of the relevant target genes, thus preventing kidney fibrosis. TCF/LEF, T cell factor/lymphoid enhancer factor.

**Figure 3:**
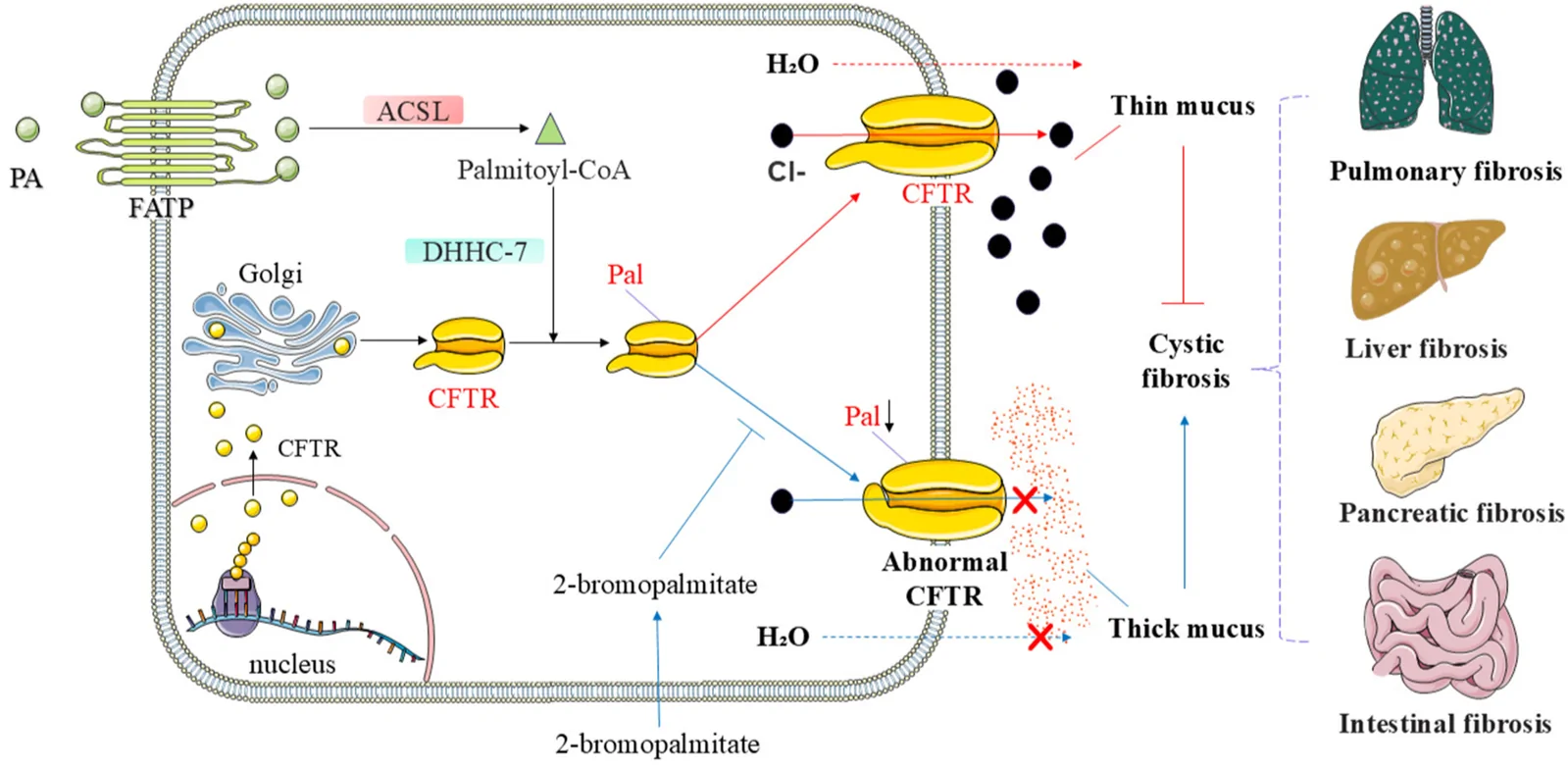
Palmitoylation in cystic fibrosis. DHHC-7 mediates CFTR palmitoylation, promoting its maturation and transport, and regulating chloride ion transmembrane movement. In contrast, 2-bromopalmitate inhibits CFTR palmitoylation, preventing chloride ion and water efflux. This causes abnormal extracellular mucus, leading to cystic fibrosis and potential fibrosis in multiple tissues and organs. CFTR, cystic fibrosis transmembrane conductance regulator; FATP, fatty acid transport protein; ACSL, long-chain acyl-CoA synthetase.

The cyclic GMP-AMP synthase (cGAS)–stimulator of interferon genes (STING) signaling pathway modulates a broad spectrum of cellular biological processes.^62^ Mounting preclinical and clinical evidence has corroborated that hyperactivation of the cGAS–STING pathway is closely associated with the pathogenesis of multiple fibrotic disorders, via mechanisms including fibroblast activation, enhanced pro-inflammatory cytokine expression, and ECM deposition.^63^, ^64^, ^65^, ^66^, ^67^ Currently, researchers have devised a strategy involving nitroalkylation of critical residues within STING, which suppresses STING palmitoylation; this, in turn, abrogates type I interferon secretion and fine-tunes inflammatory responses.^68^ Building on this mechanistic insight, targeted inhibition of STING palmitoylation sites to dampen STING-mediated innate immune response activation holds promise for attenuating the progression of fibrotic lesions.^69^ Collectively, targeting STING palmitoylation sites to modulate cGAS–STING signaling represents a promising novel therapeutic paradigm for the management of fibrotic diseases.

As a common lipid PTM, palmitoylation coregulates multi-organ fibrosis by modulating key signaling pathways (*e.g.*, β-catenin degradation, cGAS–STING activation) and downstream cellular processes, thus serving as a promising target for antifibrotic therapy. The dynamic homeostasis of protein palmitoylation is coordinately regulated by palmitoyltransferases (DHHC family) and depalmitoylases (APT family). Inhibitors targeting these enzymes, such as 2-bromopalmitate (a palmitoyltransferase inhibitor), have demonstrated anti-fibrotic efficacy in preclinical models of lung fibrosis.^70^ However, no palmitoylation-targeted therapy has advanced to clinical trials. A major pharmacological challenge is the potential for on-target toxicity, as DHHC and APT enzymes are ubiquitously involved in normal cellular signaling. Therefore, developing agents that selectively target fibrosis-specific enzyme isoforms is crucial for minimizing adverse effects and achieving therapeutic relevance.

### Crotonylation

Research on protein crotonylation has focused mainly on stem cell biology and tumorigenesis.^71^, ^72^, ^73^, ^74^ Fibrosis-related studies remain scarce, yet its significance is far from negligible. Cai et al investigated crotonylation site and protein level alterations in cardiac tissues post-ischemia–reperfusion injury. They found that over-crotonylation of myofilament and mitochondrial proteins markedly reduced myocardial fibrosis.^75^ Unfortunately, the article lacks specific details on the mechanisms underlying fibrosis reduction. These may involve increased crotonylation expression, decreased cardiac apoptosis, and reduced release of fibrosis-related inflammatory factors. Similarly, in fibrotic kidneys, a comprehensive analysis of chromatin immunoprecipitation sequencing and RNA sequencing has revealed that interleukin-1 beta (IL-1β: a pro-inflammatory cytokine regulating inflammation and immune responses), which is regulated by H3K9cr, plays a pivotal role in the fibrosis process. Furthermore, knockout of the acyl-CoA synthetase short-chain family member 2 (*ACSS2*) gene and pharmacological inhibition can reduce H3K9cr-mediated IL-1β expression, thereby alleviating renal fibrosis^76^ (Fig. 2). This suggests that ACSS2 represents a promising target for drug treatment of fibrosis.

Vilarinho et al identified a congenital acyl-CoA oxidase 2 (ACOX2)-deficient patient with hepatic fibrosis and other manifestations;^77^ notably, ACOX2, a key crotonylation regulator, is closely associated with hepatic fibrosis. Zhang et al reported down-regulated non-histone crotonylation in mitochondria/peroxisomes of fibrotic livers from ACOX2^−/−^ mice,^78^ while Chen et al linked histone crotonylation down-regulation (especially H2BK12 and H3K18) to hepatic fibrosis progression^79^ (Fig. 4). Additionally, ACOX2 protects against renal fibrosis,^80^ highlighting the interplay among ACOX2, lysine crotonylation, and renal fibrosis as a worthy research direction.

**Figure 4:**
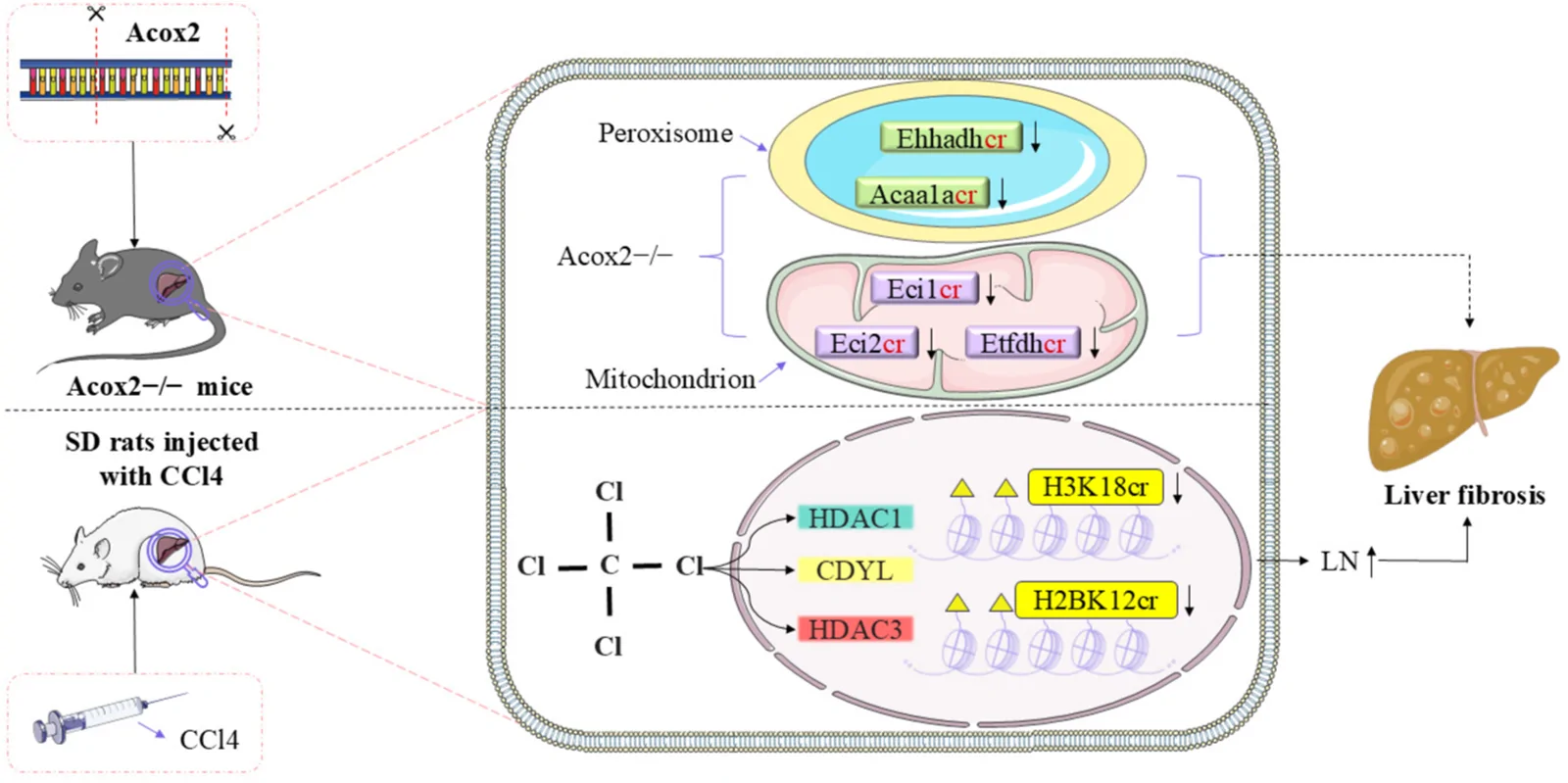
Crotonylation in liver fibrosis. In the carbon tetrachloride-induced liver fibrosis model, crotonylase removal enzymes increase, reducing histone crotonylation and triggering fibrosis-related pathways. In Acox2-knockout mice, non-histone crotonylation in peroxisomes and mitochondria is down-regulated, which may relate to liver fibrosis. SD, Sprague Dawley; HDAC, histone deacetylase; CDYL, chromodomain Y-like protein; LN, laminin.

Most studies demonstrate that histone crotonylation acts as a driver of fibrosis, while emerging evidence suggests that crotonylation dysregulation may instead represent a secondary consequence of the fibrotic process.^77^^,^^78^ Collectively, targeting crotonylation-associated regulatory enzymes (ACSS2, ACOX2) harbors substantial antifibrotic potential. However, these enzymes do not exert exclusive, specific control over crotonylation; they also modulate other post-translational modifications (*e.g.*, acetylation). Such pleiotropic activity not only hinders the safe and effective achievement of desired antifibrotic outcomes but also elevates the risk of adverse effects. Thus, precision targeting of enzyme isoforms that are specifically expressed in fibrotic tissues constitutes the core strategy for mitigating therapeutic side effects.

### Myristoylation

Protein myristoylation is primarily divided into irreversible N-terminal myristoylation and reversible lysine myristoylation. This modification mediates diverse physiological and pathological processes by regulating protein localization, stability, and signal transduction, and stands as a cutting-edge direction in fibrosis research.

McHugh et al demonstrated that treatment with N-myristoyltransferase inhibitors (NMTi) reduced fibrosis in an idiopathic pulmonary fibrosis model and led to decreases in inflammation, steatosis, and hepatic fibrosis in a non-alcoholic steatohepatitis model.^81^ The underlying mechanism may involve the reduction of ADP-ribosylation factor (ARF: a small GTPase regulating membrane trafficking and cytoskeleton) GTPase levels due to NMTi treatment, which causes the diffusion of the Golgi in senescent cells, the accumulation of intracellularly secreted cytokines, and the activation of the unfolded protein response (UPR: a cellular response to ER stress, handling unfolded/misfolded proteins to maintain ER homeostasis). This sequence of events ultimately inhibits the senescence-associated secretory phenotype (SASP: factors secreted by senescent cells, driving inflammation/tissue remodeling) and selectively eliminates senescent cells, thereby achieving anti-aging, anti-inflammatory, and anti-fibrotic effects. Furthermore, reversible lysine myristoylation regulates G protein-coupled receptor (GPCR) signaling. It facilitates beta-adrenergic receptor (β-AR) localization in lipid raft membrane microdomains. This localization promotes protein kinase A (PKA: a cAMP-activated serine/threonine kinase that phosphorylates targets to regulate cell processes) activation and downstream signaling.^82^ Studies have confirmed that abnormalities of this pathway are involved in cardiac fibrosis,^83^ cystic fibrosis,^84^ and masticatory muscle fibrosis.^85^ In summary, myristoylation acts as a key driver of the initiation and progression of fibrosis.

As a key type of lipidation, myristoylation mediates downstream effects including inflammation suppression, senescent cell clearance, and reduced ECM deposition via pathways such as the lysine myristoylation–GPCR-β–AR axis, thereby exerting a pivotal regulatory role in multi-organ fibrosis. NMT inhibitors exhibit promising antifibrotic potential; however, clinical trials of such inhibitors have thus far focused primarily on oncology indications.^86^ Additionally, no studies have yet addressed the crosstalk between myristoylation and other PTMs in the context of fibrosis.^87^

### β-hydroxybutyrylation

β-Hydroxybutyrylation plays a critical role in diverse physiological and pathological processes.^88^ Initially, a focused search using the keywords "β-hydroxybutyrylation” and “fibrosis” revealed a limited number of relevant studies. A study discovered that the up-regulation of matrix metalloproteinase 2 (MMP2: a matrix metalloproteinase that degrades ECM), mediated by H3K9bhb, antagonizes glomerulosclerosis in diabetic rats^89^ (Fig. 2). Based on the close association between β-hydroxybutyrate (BHB) and β-hydroxybutyrylation, existing evidence mainly comes from preclinical studies on BHB regulating fibrosis. Consequently, this review focuses on the relationship between BHB and fibrosis. Wang et al have demonstrated that BHB influences adipose precursor cells, inhibiting adipose tissue fibrosis by promoting the differentiation of beige adipocytes.^90^ Additionally, BHB has been shown to inhibit renal cyst growth in patients with polycystic kidney disease by improving renal fibrosis.^91^ While most studies suggest that BHB is beneficial to liver tissue, Liao et al reported that BHB may exacerbate liver fibrosis induced by carbon tetrachloride and thioacetamide.^92^ Furthermore, the role of BHB in cardiac tissue remains contentious. BHB has been found to ameliorate myocardial fibrosis in diabetic rats^93^ and to reduce myocardial fibrosis induced by doxorubicin.^94^ Conversely, Xu et al observed that BHB inhibited mitochondrial biogenesis and induced cardiac fibrosis experimentally.^95^ It should be noted that most of the above studies did not explore the direct role of β-hydroxybutyrylation in these processes.

β-Hydroxybutyrylation regulates ECM remodeling through the H3K9bhb–MMP2 pathway and exerts an antagonistic role in renal fibrosis; its substrate BHB has tissue-specific and state-dependent effects on multi-organ fibrosis, showing dual effects of promoting or inhibiting fibrosis. Although BHB is closely related to β-hydroxybutyrylation, most existing studies focus on BHB itself, and the direct regulatory role of β-hydroxybutyrylation in fibrosis and its crosstalk mechanism with other PTMs has not been clarified. Moving forward, further investigations are required to clarify the core role of β-hydroxybutyrylation in fibrosis and advance the development of relevant targeted therapies.

### Malonylation

While malonylation modifications may not be as pronounced as other novel modifications, their importance in immunity and metabolism should not be underestimated. It is well-established that tumor necrosis factor (TNF: a pro-inflammatory cytokine regulating inflammation and cell death) and IL-1β are key upstream mediators contributing to fibrosis. Notably, studies have shown that malonylation modifications are intricately linked to these inflammatory factors. Galván-Peña et al found that in macrophages stimulated by lipopolysaccharide (a Gram-negative bacterial cell wall component that induces immune responses), the K213 position of glyceraldehyde-3-phosphate dehydrogenase (GAPDH: a key enzyme in glycolysis, catalyzing glyceraldehyde-3-phosphate oxidation) undergoes malonylation, enhancing its catalytic activity.^96^ This increase in activity facilitates the glycolytic process and elevates IL-1β levels. Concurrently, the binding affinity to *TNFα* mRNA is reduced, leading to up-regulation of TNFα expression. Thus, these two processes may synergistically contribute to the progression of inflammation and fibrosis.

SIRT5 is a novel deacylase closely associated with lysine desuccinylation and desmalonylation.^97^ Mice with *SIRT5* overexpression exhibited attenuated myocardial fibrosis compared with wild-type mice.^98^ However, this article fails to elucidate the mechanism underlying these modifications, leaving the roles of succinylation and malonylation uncertain. Furthermore, sepsis-induced myocardial dysfunction is a serious complication of sepsis. It may cause abnormal myocardial contraction, reduced ejection fraction, myocardial hypertrophy, remodeling, and myocardial fibrosis. She et al^99^ demonstrated that malonylation of the voltage-dependent anion channel 2 (VDAC2: a voltage-dependent transmembrane channel on mitochondrial outer membrane, mediating metabolite transport) occurs in sepsis-induced myocardial dysfunction, where post-sepsis VDAC2 malonylation contributes to myocardial injury through mitochondria-associated iron death. Notably, myocardial injury was alleviated by targeted inhibition of malonyl-CoA.

Malonylation plays a core pro-inflammatory and pro-injury role in fibrotic progression by regulating GAPDH-mediated inflammatory factor release and VDAC2-related mitochondrial ferroptosis, thereby acting as a driver of fibrosis. Targeting malonylation or its regulatory enzyme SIRT5 holds potential antifibrotic therapeutic value. However, numerous critical questions remain to be addressed: the precise molecular mechanisms underlying malonylation-mediated fibrosis regulation are incompletely elucidated; the intricate PTM crosstalk network mediated by SIRT5 is not fully characterized; and highly specific small-molecule modulators targeting this pathway are currently lacking.

### Succinylation

Succinyl-CoA serves as a key substrate for succinylation. Given its unique role in glycolipid metabolism, most studies on succinylation have been conducted from the perspective of energy metabolism.^100^ Abnormal brain glucose metabolism is a hallmark of Alzheimer's disease neuropathology. Yang et al discovered that succinylation, a metabolism-associated PTM of proteins, may provide a critical link between metabolic abnormalities and the pathology of Alzheimer's disease.^101^ Upon quantifying cellular protein succinylation, they observed significantly elevated levels of succinylation at tau K311 and amyloid precursor protein (APP: a transmembrane protein; abnormal cleavage produces amyloid-β) K612. Ultimately, the researchers have concluded that succinylation modifications promote β-amyloid (Aβ: a peptide from abnormal APP cleavage; aggregates into plaques in Alzheimer's disease) deposition, tau fibrillation, and other processes associated with the pathogenesis of Alzheimer's disease. Additionally, succinylation participates in hepatic fibrosis by regulating the modification of protein arginine methyltransferase 5 (PRMT5) at the K387 residue: SIRT7-mediated desuccinylation stabilizes PRMT5, which in turn inhibits the cGAS–STING pathway and suppresses hepatic stellate cell senescence, ultimately promoting hepatic fibrosis.^102^ As discussed above, succinylation acts primarily as a driving factor in the progression of fibrosis; yet, emerging evidence indicates that it may also serve as a consequence of fibrosis. This is exemplified by the observation that the level of succinylation of mitochondrial proteins is markedly elevated in the lung tissues of patients with idiopathic pulmonary fibrosis.^103^

Existing studies suggest that succinylation may participate in fibrotic progression via substrate-mediated indirect regulation. A plausible mechanism is that succinate, as the precursor of succinyl-CoA, can modulate global succinylation levels through its concentration fluctuations.^104^ Notably, growing evidence indicates that succinate promotes fibrotic progression. It is thought to drive fibrosis in the liver, lung, intestine, and other organs by elevating succinylation levels, thereby regulating downstream cellular effects, including fibroblast activation and ECM deposition.^105^, ^106^, ^107^

Targeting succinylation modification or its substrate metabolic pathway represents a promising strategy for antifibrotic therapy, but related research remains in the preclinical stage. For instance, Oroxylin A-mediated SIRT7 inhibition promotes PRMT5 succinylation and degradation, which reversely activates the cGAS–STING pathway to induce hepatic stellate cell senescence, ultimately exerting antifibrotic effects against hepatic fibrosis.^105^ In terms of side effects, succinylation of regulatory enzymes is widely involved in energy metabolism and gene transcription regulation in normal cells; thus, non-specific interventions may induce adverse effects such as mitochondrial dysfunction and metabolic disorders. In summary, research on succinylation and fibrosis still has a long way to go to advance from preclinical investigations to clinical translation.

## Conclusions and prospects

With the advancement of histological techniques, an increasing number of novel PTMs have been discovered. Meanwhile, the role of PTMs in fibrosis as a key mechanism for regulating protein function has also been revealed. Han et al summarized the roles of SUMOylation and NEDDylation in fibrosis,^108^ and Zhou et al summarized the effects of N6-methyladenosine modification in fibrosis.^109^ A core insight emerging from this synthesis is that these novel PTMs mediate fibrosis through intricate crosstalk between metabolic pathways and epigenetic regulation (Table 1). For instance, lactylation links glycolytic lactate accumulation to the activation of pro-fibrotic signaling cascades (*e.g.*, TGF-β/Smad2, NF-κB); palmitoylation dynamically modulates the stability and function of key molecules like β-catenin and CFTR to regulate fibrotic responses. Collectively, these findings establish novel PTMs as integral components of the fibrogenic regulatory network, expanding our understanding of fibrosis pathogenesis beyond classical PTMs.

**Table 1:** Summary of post-translational modifications in fibrosis.

Post-translational modification	Modifying enzyme	Sites	Mechanism	Results	Reference
Lactylation	Writer: p300/CBP, ARSS1/2	Snail1la↑	Endothelial-to-mesenchymal transition, TGF-β/Smad2↑	Cardiac fibrosis↑	42
	Eraser: HDAC1–3, SIRT1–3	H3K18la↑	FN1, SERPINE1↑	Placental fibrosis↑	43
		Kla↑	TGF-β/Smad2/3, VEGFA/ERK, endothelial-to-mesenchymal transition↑	Pulmonary fibrosis↑	49
		H4K12la↑	NF-kB↑	Kidney fibrosis↑	44
		H3K18la↑	Collagens, a-SMA↑	Liver fibrosis↑	50
		SORBS3la↑	MCL-BAX/BAK↑	Liver fibrosis↑	51
		H3K18la↑	Endothelial-to-mesenchymal transition, NLRP3↑	Kidney fibrosis↑	45
		H3K18la, Ezrinla↑	RhoA/ROCK↑	Kidney fibrosis↑	46,47
		USP2la↑	TGF-β1↑	Kidney fibrosis↑	52
		Kla↑	Extracellular matrix, a-SMA↑	Pulmonary fibrosis↑	54
Palmitoylation	Writer: DHHCs	β-catenin palmitoylation↓	β-catenin, FN↑	Kidney fibrosis↑	57
	Eraser: APTs, ABHD17	CFTR palmitoylation↓	CFTR trafficking and maturation↓	Cystic fibrosis↑	59
		HK1 palmitoylation↑	HK secretion and hijacking↑	Liver fibrosis↑	60
		BECN1 S-palmitoylation↑	Hepatic stellate cell ferroptosis↑	Liver fibrosis↓	58
Crotonylation	Writer: p300/CBP, ACSS2	Ehhadhcr↓	DNA damage↑	Liver fibrosis↑	78
	Eraser: HDAC1–3, CDYL	H2BK12cr↓, H3K18cr↓	PC-III, HA, LN↑	Liver fibrosis↑	79
		H3K9cr↑	IL-1β↑	Kidney fibrosis↑	76
Myristoylation	Writer: NMT1, NMT2	Protein myristoylation↑	Cxcl5, α-SMA↑	Pulmonary fibrosis↑	81
	Eraser: -	Protein myristoylation↑	Collagens↑	Liver fibrosis↑	81
β-hydroxybutyrylation	Writer: p300/CBP	H3K9bnb↑	MMP2↑	Kidney fibrosis↓	89
	Eraser: HDAC1–3, SIRT1–3				
Malonylation	Writer: p300/CBP	VDAC2 malonylation↑	Ferroptosis↑	Myocardial fibrosis↑	99
	Eraser: SIRT5				
Succinylation	Writer: P300/CBP	PRMT5 succinylation↑	Hepatic stellate cell senescence↑	Liver fibrosis↓	102
	Eraser: SIRT5, SIRT7				

Accumulating evidence from recent studies indicates that the anti-fibrotic mechanisms of action of certain therapeutic agents are intricately linked to PTMs (Fig. 5). Notably, nintedanib, gefitinib, and foretinib have been shown to suppress pulmonary fibrosis through regulating the phosphorylation of key target factors.^110^^,^^111^ These findings collectively underscore that targeting PTMs involved in the pathogenic cascades of fibrotic diseases constitutes a promising therapeutic approach. Specifically, emerging evidence demonstrates that inhibition of neddylation effectively mitigates renal fibrosis;^112^ sorafenib ameliorates hepatic fibrosis via modulating crotonylation of critical substrates;^65^ and targeting the N6-methyladenosine modification of insulin-like growth factor 2 (IGF2) mRNA-binding protein 2 (IGF2BP2) exhibits considerable potential as a targeted therapeutic strategy for hepatic fibrosis.^113^ Interestingly, researchers have also summarized the role of natural medicines in PTM-regulated fibrotic nodes, providing a novel perspective for the development of antifibrotic drugs.^114^

**Figure 5:**
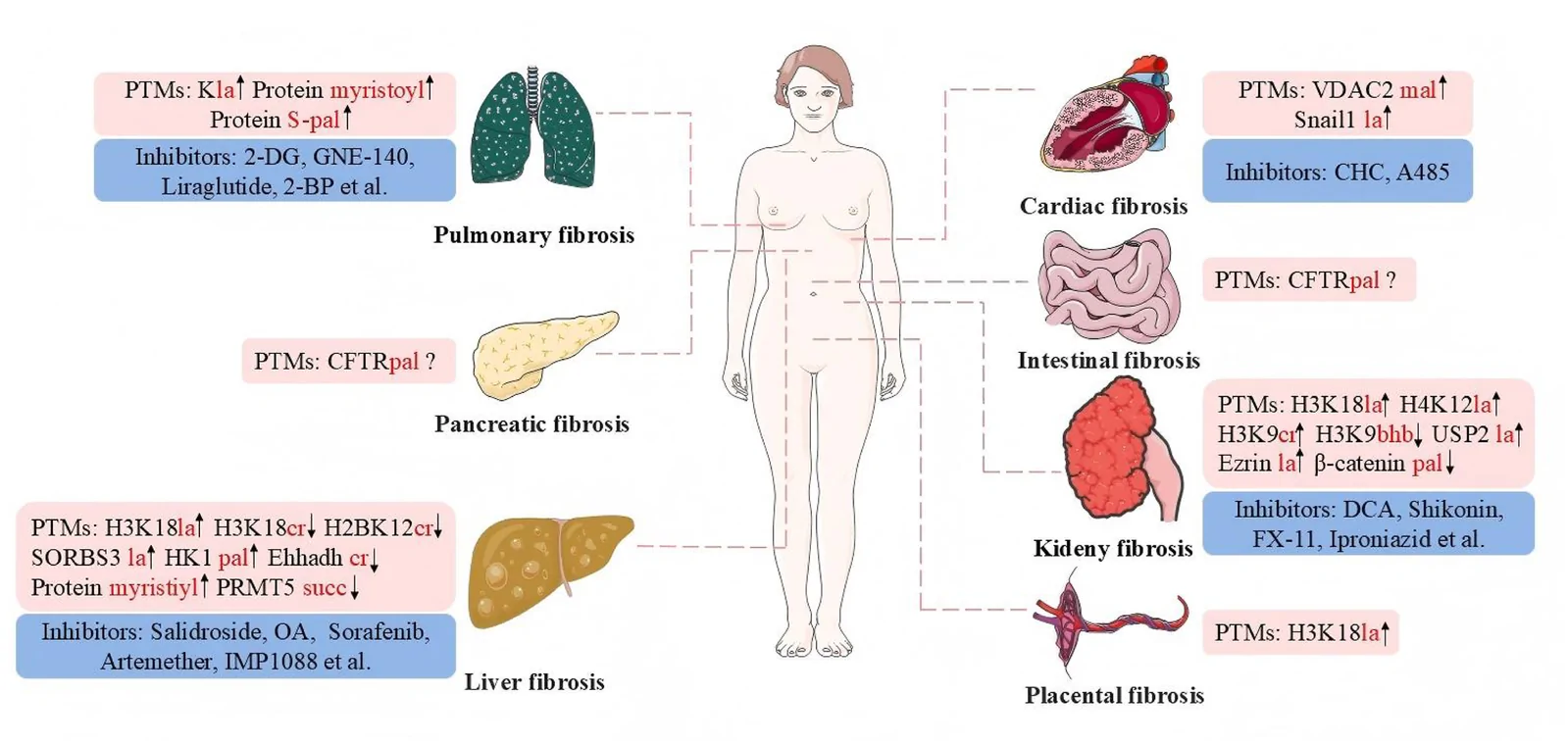
Post-translational modifications (PTMs) in fibrosis and their potential therapeutic strategies. 2-DG, 2-deoxy-d-glucose; CHC, α-cyano-4-hydroxycinnamic acid; 3PO, 3-(3-pyridinyl)-1-(4-pyridinyl)-2-propen-1-one; DCA, dichloroacetate; Ox, oxamate.

However, several challenges exist in this field. First, the mechanisms underlying most novel PTMs in fibrosis remain incompletely elucidated. Second, the tissue-specific and context-dependent effects of PTMs remain poorly understood, with limited clarity on the upstream metabolic signals or microenvironmental factors that dictate these divergent outcomes. Third, the crosstalk between different novel PTMs and their interactions with classical PTMs have not been systematically explored. Fourth, the enzyme systems governing many novel PTMs are incompletely characterized, hindering the development of targeted interventions. Finally, the translational potential of PTM-targeted therapies is constrained by a lack of clinical data linking PTM levels to disease severity or treatment response in fibrotic patients.

To address these gaps, future research should prioritize the following directions. First, state-of-the-art techniques and experimental approaches—including site-directed mutagenesis, genetic code expansion, chemical mutagenesis, and dialAGE—are required to dissect their functional roles and elucidate their implications in fibrogenesis.^115^, ^116^, ^117^ Second, studies focusing on PTM crosstalk—both between novel PTMs and with classical modifications—will provide a more comprehensive understanding of the regulatory networks driving fibrosis. Third, the development of highly specific small-molecule inhibitors or activators targeting PTM-related enzymes is critical; these agents should be evaluated in preclinical models of organ-specific fibrosis to assess efficacy and safety. Fourth, clinical translational studies are essential to validate PTMs as diagnostic biomarkers and to test the efficacy of PTM-targeted therapies in clinical trials, particularly in combination with existing anti-fibrotic agents to enhance therapeutic outcomes. Additionally, exploring the role of PTMs in reversible stages of fibrosis may uncover strategies to halt or reverse disease progression, addressing the unmet clinical need for curative therapies.

In conclusion, novel PTMs play a key role in fibrosis development through metabolism–epigenetic crosstalk, and targeting novel PTMs represents a promising anti-fibrotic strategy. While significant challenges remain in understanding their precise mechanisms and translating findings to the clinic, continued research in this field holds great potential to revolutionize the diagnosis and treatment of fibrotic diseases, ultimately reducing the global health burden associated with these debilitating conditions.

## CRediT authorship contribution statement

**Qianshi Wu:** Writing – original draft. **Li Luo:** Writing – original draft. **Yanqun Li:** Writing – original draft. **Jidi Wu:** Writing – original draft. **Junle Li:** Writing – original draft. **Wanhong He:** Writing – original draft. **Yulin Mou:** Writing – original draft. **Shiying Qu:** Writing – original draft. **Yanqi Lei:** Writing – original draft. **Wei Huang:** Writing – review & editing. **Ling Cao:** Writing – review & editing.

## Data availability

Data will be made available on request.

## Funding

This work was supported by grants from the 10.13039/501100001809National Natural Science Foundation of China (No. 82170834, U22A20286), the 10.13039/501100018537National Science and Technology Major Project (China) (No. 2024ZD0531901), the 10.13039/501100004829Science and Technology Department of Sichuan Province, China (No. 2024YFFK0081), Health Commission of Sichuan Province Medical Science and Technology Program (China) (No. 24CXTD02), the Office of Science Technology and Talent Work of Luzhou, Sichuan, China (No. 2025JBH003), Clinical Medicine Special Project of Southwest Medical University (China) (No. 2024LCYXZX12), the Project of Southwest Medical University (China) (No. 2022QN059, YJG202291), and Graduate Education and Teaching Program of Southwest Medical University (China) (No. YJG202291, ZYTS-29).

## Conflict of interests

The authors declare that they have no known competing financial interests or personal relationships that could have appeared to influence the work reported in this paper.

## References

[1] Vos T., Lim S.S., Abbafati C. (2020). Global burden of 369 diseases and injuries in 204 countries and territories, 1990–2019: a systematic analysis for the global burden of disease study 2019. Lancet.

[2] Zhao X., Kwan J.Y.Y., Yip K., Liu P.P., Liu F.F. (2020). Targeting metabolic dysregulation for fibrosis therapy. Nat Rev Drug Discov.

[3] Strahl B.D., Allis C.D. (2000). The language of covalent histone modifications. Nature.

[4] Kouzarides T. (2007). Chromatin modifications and their function. Cell.

[5] Allis C.D., Jenuwein T. (2016). The molecular hallmarks of epigenetic control. Nat Rev Genet.

[6] Hunter T. (2012). Why nature chose phosphate to modify proteins. Philos Trans R Soc Lond B Biol Sci.

[7] Olsen J.V., Blagoev B., Gnad F. (2006). Global, *in vivo*, and site-specific phosphorylation dynamics in signaling networks. Cell.

[8] Walsh C.T., Garneau-Tsodikova S., Gatto G.J. (2005). Protein posttranslational modifications: the chemistry of proteome diversifications. Angew Chem Int Ed Engl.

[9] Hay R.T. (2005). SUMO: a history of modification. Mol Cell.

[10] Olsen J.V., Mann M. (2013). Status of large-scale analysis of post-translational modifications by mass spectrometry. Mol Cell Proteomics.

[11] Zhang D., Tang Z., Huang H. (2019). Metabolic regulation of gene expression by histone lactylation. Nature.

[12] Schmidt M.F.G., Bracha M., Schlesinger M.J. (1979). Evidence for covalent attachment of fatty acids to Sindbis virus glycoproteins. Proc Natl Acad Sci U S A.

[13] Tan M., Luo H., Lee S. (2011). Identification of 67 histone marks and histone lysine crotonylation as a new type of histone modification. Cell.

[14] Aitken A., Cohen P., Santikarn S. (1982). Identification of the NH_2_-terminal blocking group of calcineurin B as myristic acid. FEBS Lett.

[15] Xie Z., Zhang D., Chung D. (2016). Metabolic regulation of gene expression by histone lysine β-hydroxybutyrylation. Mol Cell.

[16] Peng C., Lu Z., Xie Z. (2011). The first identification of lysine malonylation substrates and its regulatory enzyme. Mol Cell Proteomics.

[17] Zhang Z., Tan M., Xie Z., Dai L., Chen Y., Zhao Y. (2011). Identification of lysine succinylation as a new post-translational modification. Nat Chem Biol.

[18] Xue T., Qiu X., Liu H. (2021). Epigenetic regulation in fibrosis progress. Pharmacol Res.

[19] Gabbiani G. (2003). The myofibroblast in wound healing and fibrocontractive diseases. J Pathol.

[20] Minton K. (2014). Preconditioning the ECM for fibrosis. Nat Rev Mol Cell Biol.

[21] Frangogiannis N.G. (2020). Transforming growth factor-β in tissue fibrosis. J Exp Med.

[22] Giarratana A.O., Prendergast C.M., Salvatore M.M., Capaccione K.M. (2024). TGF-β signaling: critical nexus of fibrogenesis and cancer. J Transl Med.

[23] Shi X., Young C.D., Zhou H., Wang X.J. (2020). Transforming growth factor-β signaling in fibrotic diseases and cancer-associated fibroblasts. Biomolecules.

[24] Biernacka A., Dobaczewski M., Frangogiannis N.G. (2011). TGF-β signaling in fibrosis. Growth Factors.

[25] Peng D., Fu M., Wang M., Wei Y., Wei X. (2022). Targeting TGF-β signal transduction for fibrosis and cancer therapy. Mol Cancer.

[26] Piersma B., Bank R.A., Boersema M. (2015). Signaling in fibrosis: Tgf-β, WNT, and YAP/TAZ converge. Front Med.

[27] Liu J., Xiao Q., Xiao J. (2022). Wnt/β-catenin signalling: function, biological mechanisms, and therapeutic opportunities. Signal Transduct Target Ther.

[28] Akhmetshina A., Palumbo K., Dees C. (2012). Activation of canonical Wnt signalling is required for TGF-β-mediated fibrosis. Nat Commun.

[29] Bakalenko N., Kuznetsova E., Malashicheva A. (2024). The complex interplay of TGF-β and notch signaling in the pathogenesis of fibrosis. Int J Mol Sci.

[30] Zheng S., Xue C., Li S. (2024). Research progress of Chinese medicine in the regulation of liver fibrosis-related signaling pathways. Am J Chin Med.

[31] Zhang X., Xu Z., Chen Q., Zhou Z. (2024). Notch signaling regulates pulmonary fibrosis. Front Cell Dev Biol.

[32] Moreno-Yruela C., Zhang D., Wei W. (2022). Class I histone deacetylases (HDAC1-3) are histone lysine delactylases. Sci Adv.

[33] Wang J., Yang P., Yu T. (2022). Lactylation of PKM2 suppresses inflammatory metabolic adaptation in pro-inflammatory macrophages. Int J Biol Sci.

[34] Zong Z., Xie F., Wang S. (2024). Alanyl-tRNA synthetase, AARS1, is a lactate sensor and lactyltransferase that lactylates p53 and contributes to tumorigenesis. Cell.

[35] Li X.M., Yang Y., Jiang F.Q. (2024). Histone lactylation inhibits RARγ expression in macrophages to promote colorectal tumorigenesis through activation of TRAF6-IL-6-STAT3 signaling. Cell Rep.

[36] Yang H., Mo N., Tong L. (2025). Microglia lactylation in relation to central nervous system diseases. Neural Regen Res.

[37] Wu B., Liu Y., Li H. (2025). Liver as a new target organ in Alzheimer's disease: insight from cholesterol metabolism and its role in amyloid-beta clearance. Neural Regen Res.

[38] Chen Z., Wan B., Zhang H. (2024). Histone lactylation mediated by Fam172a in POMC neurons regulates energy balance. Nat Commun.

[39] Gao R., Li Y., Xu Z. (2023). Mitochondrial pyruvate carrier 1 regulates fatty acid synthase lactylation and mediates treatment of nonalcoholic fatty liver disease. Hepatology.

[40] Fan W., Wang X., Zeng S. (2023). Global lactylome reveals lactylation-dependent mechanisms underlying T_H_17 differentiation in experimental autoimmune uveitis. Sci Adv.

[41] Chen L., Zhang M., Yang X. (2024). Methyl-CpG-binding 2 K271 lactylation-mediated M2 macrophage polarization inhibits atherosclerosis. Theranostics.

[42] Fan M., Yang K., Wang X. (2023). Lactate promotes endothelial-to-mesenchymal transition via Snail1 lactylation after myocardial infarction. Sci Adv.

[43] Li X., Yang N., Wu Y. (2022). Hypoxia regulates fibrosis-related genes via histone lactylation in the placentas of patients with preeclampsia. J Hypertens.

[44] Wang Y., Li H., Jiang S. (2024). The glycolytic enzyme PFKFB3 drives kidney fibrosis through promoting histone lactylation-mediated NF-κB family activation. Kidney Int.

[45] Hu X., Chen W., Yang M., Li M., Li X., Ouyang S. (2025). IGFBP5 promotes EndoMT and renal fibrosis through H3K18 lactylation in diabetic nephropathy. Cell Mol Life Sci.

[46] Qiao J., Tan Y., Liu H. (2024). Histone H3K18 and ezrin lactylation promote renal dysfunction in sepsis-associated acute kidney injury. Adv Sci.

[47] Shi Z., Wang Q., Zhang Y., Jiang D. (2020). Extracellular vesicles produced by bone marrow mesenchymal stem cells attenuate renal fibrosis, in part by inhibiting the RhoA/ROCK pathway, in a UUO rat model. Stem Cell Res Ther.

[48] Cui H., Xie N., Banerjee S. (2021). Lung myofibroblasts promote macrophage profibrotic activity through lactate-induced histone lactylation. Am J Respir Cell Mol Biol.

[49] Li J., Zeng G., Zhang Z. (2024). Urban airborne PM_2.5_ induces pulmonary fibrosis through triggering glycolysis and subsequent modification of histone lactylation in macrophages. Ecotoxicol Environ Saf.

[50] Rho H., Terry A.R., Chronis C., Hay N. (2023). Hexokinase 2-mediated gene expression via histone lactylation is required for hepatic stellate cell activation and liver fibrosis. Cell Metab.

[51] Liu Y., Zhou R., Guo Y. (2025). Muscle-derived small extracellular vesicles induce liver fibrosis during overtraining. Cell Metab.

[52] Xiang J., Yang G., Li L. (2025). Lactate orchestrates the TGFβ pathway and ferroptosis nexus in organ fibrosis via USP2 lactylation. Commun Biol.

[53] Liang H., Xu L., Yang Y. (2025). Lactate and lactylation: novel perspectives on fibrosis pathogenesis and therapeutic directions. J Transl Med.

[54] Liu C., Zhang Q., Zhou H. (2024). GLP-1R activation attenuates the progression of pulmonary fibrosis via disrupting NLRP3 inflammasome/PFKFB3-driven glycolysis interaction and histone lactylation. J Transl Med.

[55] Flender D., Vilenne F., Adams C., Boonen K., Valkenborg D., Baggerman G. (2025). Exploring the dynamic landscape of immunopeptidomics: unravelling posttranslational modifications and navigating bioinformatics terrain. Mass Spectrom Rev.

[56] Ren H., Tang Y., Zhang D. (2025). The emerging role of protein L-lactylation in metabolic regulation and cell signalling. Nat Metab.

[57] Gu M., Jiang H., Tan M. (2023). Palmitoyltransferase DHHC9 and acyl protein thioesterase APT1 modulate renal fibrosis through regulating β-catenin palmitoylation. Nat Commun.

[58] Li M., Sun Y., Wei Y. (2025). Artemether relieves liver fibrosis by triggering ferroptosis in hepatic stellate cells via DHHC12-mediated S-palmitoylation of the BECN1 protein. Free Radic Biol Med.

[59] McClure M.L., Wen H., Fortenberry J., Hong J.S., Sorscher E.J. (2014). S-palmitoylation regulates biogenesis of core glycosylated wild-type and F508del CFTR in a post-ER compartment. Biochem J.

[60] Chen Q.T., Zhang Z.Y., Huang Q.L. (2022). HK1 from hepatic stellate cell-derived extracellular vesicles promotes progression of hepatocellular carcinoma. Nat Metab.

[61] Liang R., Kagwiria R., Zehender A. (2019). Acyltransferase skinny hedgehog regulates TGFβ-dependent fibroblast activation in SSc. Ann Rheum Dis.

[62] Chen C., Xu P. (2023). Cellular functions of cGAS-STING signaling. Trends Cell Biol.

[63] Zhang C., Hao H., Wang Y. (2023). Intercellular mitochondrial component transfer triggers ischemic cardiac fibrosis. Sci Bull.

[64] Xiao Y., Zhao C., Tai Y. (2023). STING mediates hepatocyte pyroptosis in liver fibrosis by epigenetically activating the NLRP3 inflammasome. Redox Biol.

[65] Chung K.W., Dhillon P., Huang S. (2019). Mitochondrial damage and activation of the STING pathway lead to renal inflammation and fibrosis. Cell Metab.

[66] Zhao Q., Manohar M., Wei Y., Pandol S.J., Habtezion A. (2019). STING signalling protects against chronic pancreatitis by modulating Th17 response. Gut.

[67] Xie X., Wu X., Zhao D. (2023). Fluvoxamine alleviates bleomycin-induced lung fibrosis via regulating the cGAS-STING pathway. Pharmacol Res.

[68] Hansen A.L., Buchan G.J., Rühl M. (2018). Nitro-fatty acids are formed in response to virus infection and are potent inhibitors of STING palmitoylation and signaling. Proc Natl Acad Sci U S A.

[69] Haag S.M., Gulen M.F., Reymond L. (2018). Targeting STING with covalent small-molecule inhibitors. Nature.

[70] Lee C.M., Jarrell Z.R., Lee H.Y. (2024). Protein S-palmitoylation enhances profibrotic signaling in response to cadmium. Toxicol Appl Pharmacol.

[71] Fang Y., Xu X., Ding J. (2021). Histone crotonylation promotes mesoendodermal commitment of human embryonic stem cells. Cell Stem Cell.

[72] Liu S., Yu H., Liu Y. (2017). Chromodomain protein CDYL acts as a crotonyl-CoA hydratase to regulate histone crotonylation and spermatogenesis. Mol Cell.

[73] Liu Y., Li M., Fan M. (2019). Chromodomain Y-like protein-mediated histone crotonylation regulates stress-induced depressive behaviors. Biol Psychiatry.

[74] Wan J., Liu H., Ming L. (2019). Lysine crotonylation is involved in hepatocellular carcinoma progression. Biomed Pharmacother.

[75] Cai W., Xu D., Zeng C. (2022). Modulating lysine crotonylation in cardiomyocytes improves myocardial outcomes. Circ Res.

[76] Li L., Xiang T., Guo J. (2024). Inhibition of ACSS2-mediated histone crotonylation alleviates kidney fibrosis via IL-1β-dependent macrophage activation and tubular cell senescence. Nat Commun.

[77] Vilarinho S., Sari S., Mazzacuva F. (2016). *ACOX2*deficiency: a disorder of bile acid synthesis with transaminase elevation, liver fibrosis, ataxia, and cognitive impairment. Proc Natl Acad Sci U S A.

[78] Zhang Y., Chen Y., Zhang Z. (2022). Acox2 is a regulator of lysine crotonylation that mediates hepatic metabolic homeostasis in mice. Cell Death Dis.

[79] Chen X.F., Ji S. (2022). Sorafenib attenuates fibrotic hepatic injury through mediating lysine crotonylation. Drug Des Devel Ther.

[80] Zeng J., Huang H., Zhang Y. (2023). Dapagliflozin alleviates renal fibrosis in a mouse model of adenine-induced renal injury by inhibiting TGF-β1/MAPK mediated mitochondrial damage. Front Pharmacol.

[81] McHugh D., Sun B., Gutierrez-Muñoz C. (2023). COPI vesicle formation and N-myristoylation are targetable vulnerabilities of senescent cells. Nat Cell Biol.

[82] Bagchi R.A., Robinson E.L., Hu T. (2022). Reversible lysine fatty acylation of an anchoring protein mediates adipocyte adrenergic signaling. Proc Natl Acad Sci U S A.

[83] Xiao H., Li H., Wang J.J. (2018). IL-18 cleavage triggers cardiac inflammation and fibrosis upon β-adrenergic insult. Eur Heart J.

[84] Ito A., Ohnuki Y., Suita K. (2019). Role of β-adrenergic signaling in masseter muscle. PLoS One.

[85] Bossard F., Silantieff E., Lavazais-Blancou E. (2011). β1, β2, and β3 adrenoceptors and Na^+^/H^+^ exchanger regulatory factor 1 expression in human bronchi and their modifications in cystic fibrosis. Am J Respir Cell Mol Biol.

[86] Sangha R., Jamal R., Spratlin J. (2024). A first-in-human phase I trial of daily oral zelenirstat, a N-myristoyltransferase inhibitor, in patients with advanced solid tumors and relapsed/refractory B-cell lymphomas. Invest New Drugs.

[87] Yuan M., Song Z.H., Ying M.D. (2020). N-myristoylation: from cell biology to translational medicine. Acta Pharmacol Sin.

[88] Zhou T., Cheng X., He Y. (2022). Function and mechanism of histone β-hydroxybutyrylation in health and disease. Front Immunol.

[89] Luo W., Yu Y., Wang H. (2020). Up-regulation of MMP-2 by histone H3K9 β-hydroxybutyrylation to antagonize glomerulosclerosis in diabetic rat. Acta Diabetol.

[90] Wang W., Ishibashi J., Trefely S. (2019). A PRDM16-driven metabolic signal from adipocytes regulates precursor cell fate. Cell Metab.

[91] Torres J.A., Kruger S.L., Broderick C. (2019). Ketosis ameliorates renal cyst growth in polycystic kidney disease. Cell Metab.

[92] Liao Y.J., Wang Y.H., Wu C.Y., Hsu F.Y., Chien C.Y., Lee Y.C. (2021). Ketogenic diet enhances the cholesterol accumulation in liver and augments the severity of CCl_4_ and TAA-induced liver fibrosis in mice. Int J Mol Sci.

[93] Trang N.N., Lee T.W., Kao Y.H., Chao T., Lee T.I., Chen Y.J. (2023). Ketogenic diet modulates cardiac metabolic dysregulation in streptozocin-induced diabetic rats. J Nutr Biochem.

[94] Liu Y., Wei X., Wu M., Xu J., Xu B., Kang L. (2021). Cardioprotective roles of β-hydroxybutyrate against doxorubicin induced cardiotoxicity. Front Pharmacol.

[95] Xu S., Tao H., Cao W. (2021). Ketogenic diets inhibit mitochondrial biogenesis and induce cardiac fibrosis. Signal Transduct Target Ther.

[96] Galván-Peña S., Carroll R.G., Newman C. (2019). Malonylation of GAPDH is an inflammatory signal in macrophages. Nat Commun.

[97] Nishida Y., Rardin M.J., Carrico C. (2015). SIRT5 regulates both cytosolic and mitochondrial protein malonylation with glycolysis as a major target. Mol Cell.

[98] Zhou B., Xiao M., Hu H. (2021). Cardioprotective role of SIRT5 in response to acute ischemia through a novel liver-cardiac crosstalk mechanism. Front Cell Dev Biol.

[99] She H., Tan L., Du Y. (2023). VDAC2 malonylation participates in sepsis-induced myocardial dysfunction via mitochondrial-related ferroptosis. Int J Biol Sci.

[100] Gut P., Matilainen S., Meyer J.G. (2020). SUCLA2 mutations cause global protein succinylation contributing to the pathomechanism of a hereditary mitochondrial disease. Nat Commun.

[101] Yang Y., Tapias V., Acosta D. (2022). Altered succinylation of mitochondrial proteins, APP and tau in Alzheimer's disease. Nat Commun.

[102] Wang J., Zhang F., Shao J. (2025). Dual roles of SIRT7 inhibition by oroxylin a reprogram HSCs fate: PRMT5 succinylation-driven senescence and ecto-calreticulin-dependent NK cell immune clearance in liver fibrosis. Research (Wash D C).

[103] Zhao Y., Hou W., Yang L. (2025). Higher mitochondrial protein succinylation detected in lung tissues of idiopathic pulmonary fibrosis patients. J Proteomics.

[104] Zhang X., Liu J., Cheng Y. (2023). Metabolic enzyme Suclg2 maintains tolerogenicity of regulatory dendritic cells diffDCs by suppressing Lactb succinylation. J Autoimmun.

[105] Mills E.L., Harmon C., Jedrychowski M.P. (2021). UCP1 governs liver extracellular succinate and inflammatory pathogenesis. Nat Metab.

[106] Macias-Ceja D.C., Ortiz-Masiá D., Salvador P. (2019). Succinate receptor mediates intestinal inflammation and fibrosis. Mucosal Immunol.

[107] Rajesh R., Mooslechner A.A., Schweighofer H. (2025). Succinate aggravates pulmonary fibrosis through the succinate/SUCNR1 axis. Am J Physiol Lung Cell Mol Physiol.

[108] Han J., Wu J., Kou W.T. (2024). New insights into SUMOylation and NEDDylation in fibrosis. Front Pharmacol.

[109] Zhou Y., Jian N., Jiang C., Wang J. (2024). m^6^A modification in non-coding RNAs: mechanisms and potential therapeutic implications in fibrosis. Biomed Pharmacother.

[110] Tu J., Xu H., Ma L. (2022). Nintedanib enhances the efficacy of PD-L1 blockade by upregulating MHC-I and PD-L1 expression in tumor cells. Theranostics.

[111] Wang M., Zhang Z., Liu J. (2022). Gefitinib and fostamatinib target EGFR and SYK to attenuate silicosis: a multi-omics study with drug exploration. Signal Transduct Target Ther.

[112] Li X.Q., Jin B., Liu S.X. (2025). Neddylation of RhoA impairs its protein degradation and promotes renal interstitial fibrosis progression in diabetic nephropathy. Acta Pharmacol Sin.

[113] Zhou Y., Yan J., Huang H. (2024). The m^6^A reader IGF_2_BP_2_ regulates glycolytic metabolism and mediates histone lactylation to enhance hepatic stellate cell activation and liver fibrosis. Cell Death Dis.

[114] Niu Q., Mou Y., Wang K., Dong H., Zeng Z., Li H. (2025). Natural medicines for treating liver fibrosis by modulating post-translational modifications. Front Pharmacol.

[115] Gan Q., Fan C. (2024). Orthogonal translation for site-specific installation of post-translational modifications. Chem Rev.

[116] Ablat G., Lawton N., Alam R. (2024). Kinetic resolution of epimeric proteins enables stereoselective chemical mutagenesis. J Am Chem Soc.

[117] Martin M.S., Bayaraa N., Fox B.T., Lin Y.S., Scheck R.A. (2025). Predictable modulation of a spontaneous post-translational modification in living cells. Angew Chem Int Ed Engl.

